# Bedford and Its Medical Institutions

**Published:** 1893-02-04

**Authors:** 


### THE INSTITUTIONAL WORKSHOP.

### WITHIN THE HOSPITALS.

### BEDFORD AND ITS MEDICAL INSTITUTIONS.

BY OUR SPECIAL COMMISSIONER.

Bedford, with a population of less than 30,000, has a habit of duplicating its medical institutions in a way which would be amusing if it were not so manifestly undesirable. Thus there are two Provident Dispensaries, each of which has attached to it a nurses' institute, and neither of these institutes has its nurses trained at the Royal Infirmary.

BEDFORD PROVIDENT DISPENSARY.

This institution was established 19 years ago. The number of members is not stated, but about 40,000 prescriptions are dispensed annually, and upwards of £700 is distributed among the medical officers. The benefits of this institution are confined to members of the working classes, their wives and families, whose incomes do not average more than 30s. per week. They pay an entrance fee of 1s. No family is required to pay more than 3d. a week for parents and children under 14 years of age. Adults pay 1d. per week, children ½d. If the parents' subscriptions are three months in arrear they cease to be members, and are subject to a fine of 2d. when in arrear for three weeks, and of 3d. when the period extends to six weeks. To obtain readmission, an entrance fee of 5s. has to be forthcoming. If any of the family join who at the time are actually in need of medical attendance, they pay 7s. 6d. extra. Each member selects his own surgeon, and is at liberty to change him at the end of the current quarter; 7s. 6d. is charged for the services of a midwife, and in cases of difficulty the medical man attending the family gives his services gratuitously. Any members desiring the services of a medical man must make their own private arrangements, independently of the dispensary. A payment of 5s. a year entitles domestic servants to medical attendance and medicine at the dispensary, but a further fee is charged if they are visited at the houses of their employers. There are seven medical officers attached to this dispensary.

BEDFORD CENTRAL PROVIDENT DISPENSARY.

About five years ago, for some reason which does not appear, this institution was started at 69, Prebend Street. It has two medical officers, and about 2,900 members, and there is no wage limit for membership. The payment for a family is 2½d. a week, or 10d. per month, which is reduced to 2d. and 8d. respectively when the father is a member of a sick benefit society. A widow with children pays 1½d. per week, or 6d. per month. Domestic servants are charged from 6s. to 8s. per annum, and are attended at the house of their masters and mistresses, who may enter their servants *ex officio* on the same terms. A change of servants during the year does not render the membership invalid. Immediate medical attendance is obtainable, on joining, by an advance payment of 3s. 6d. as an entrance fee. Female members requiring the attendance of a medical officer at their confinement, whether premature or not, must pay the fee at the time they send for him, or in advance. About £200 is divided amongst the medical officers. It is a matter for regret that there should be two institutions working in competition in a small town like Bedford, and we hope that means will be found to amalgamate them without delay.

BEDFORD HOSPITAL-TRAINED NURSES' INSTITUTE.

This institution, which occupies a portion of the Provident Dispensary buildings, has been established about three years. Twelve private nurses are employed, and they attended 131 cases in 1891. There is a district nurse attached to the Institute, who looks after such poor persons as may be recommended by the medical officer to the Bedford Provident Dispensary, and others. Miss Rawson is the honorary lady superintendent. The nurses earned £620 in 1891, and about £800 was expended in wages, management, board, uniforms, &c. There can be no doubt that it would be most desirable to attach the Nurses' Institute to the infirmary, and to make arrangements that its board should train the nurses there. This would give a much better guarantee of efficiency to those who require the services of a private nurse; it would add to the funds of both institutions, and be in every way most desirable.

THE GENERAL INFIRMARY.

The older portions of the buildings of this hospital are about ninety years old. They contain no special features. The wards are small, and open, for the most part, one into the other. The lavatory and bath accommodation is out of date, and is situated in the main buildings adjacent to the wards. The whole institution is kept in a way which reflects the greatest credit upon the management and staff. It is evidently a most comfortable place of residence for the patients, and we gathered from what we saw that good work is being done within its walls. The number of beds provided appears to be ample, and although certain structural alterations will soon be necessary, and it would be well to re-point the buildings, there is nothing to warrant a large expenditure upon a new hospital, seeing that the institution at present answers every purpose, and is evidently most efficient. It would be well if the Nurses' Institute could be amalgamated, as we hope it will be, with the infirmary, which should then erect a nurses' home large enough to accommodate the infirmary staff, and a number of probationers, as well as the private nursing staff, who would be required to attend cases at patients' homes. We congratulate the managers of the Bedford Infirmary upon its general efficiency and the patients upon having so comfortable a refuge within easy reach of their homes.

